# Risk Factors for Sporadic Shiga Toxin–producing *Escherichia coli* Infections in Children, Argentina^1 ^

**DOI:** 10.3201/eid1405.071050

**Published:** 2008-05

**Authors:** Marta Rivas, Sergio Sosa-Estani, Josefa Rangel, Maria G. Caletti, Patricia Vallés, Carlos D. Roldán, Laura Balbi, Maria C. Marsano de Mollar, Diego Amoedo, Elizabeth Miliwebsky, Isabel Chinen, Robert M. Hoekstra, Paul Mead, Patricia M. Griffin

**Affiliations:** * Instituto Nacional de Enfermedades Infecciosas, Buenos Aires, Argentina; † Centro Nacional de Endemoepidemias, Buenos Aires, Argentina; ‡ Centers for Disease Control and Prevention, Atlanta, Georgia, USA; § Hospital Nacional de Pediatría, Buenos Aires, Argentina; ¶ Hospital Pediátrico, Mendoza, Argentina; # Ministerio de Desarrollo Social y Salud, Mendoza, Argentina

**Keywords:** Shiga toxin–producing Escherichia coli, E. coli O157, hemolytic uremic syndrome (HUS), case-control study, risk factors, human, transmission, epidemiology, Argentina, research

## Abstract

We evaluated risk factors for sporadic Shiga toxin–producing *Escherichia coli* (STEC) infection among children in Argentina. We conducted a prospective case–control study in 2 sites and enrolled 150 case-patients and 299 controls. The median age of case-patients was 1.8 years; 58% were girls. Serotype O157:H7 was the most commonly isolated STEC. Exposures associated with infection included eating undercooked beef, living in or visiting a place with farm animals, and contact with a child <5 years of age with diarrhea. Protective factors included the respondent reporting that he or she always washed hands after handling raw beef and the child eating more than the median number of fruits and vegetables. Many STEC infections in children could be prevented by avoiding consumption of undercooked beef, limiting exposure to farm animals and their environment, not being exposed to children with diarrhea, and washing hands after handling raw beef.

## Abstract 2

These infections can be prevented by avoiding known risk factors.

Shiga toxin–producing *Escherichia coli* (STEC) infections cause nonbloody diarrhea, hemorrhagic colitis, and hemolytic uremic syndrome (HUS) (*1*). HUS is characterized by hemolytic anemia, thrombocytopenia, and renal failure (*2*,*3*). No specific treatment exists for HUS, and the mortality rate among children with the syndrome is 5% (*3*–*5*). *E. coli* O157:H7, which was first identified as a human pathogen in 1982 (*6*), is the STEC serotype most frequently isolated from persons with diarrhea (*7*). Other STEC serotypes can cause a similar illness. STEC has been isolated from the feces of many farm animals, including ruminants (e.g., cattle, sheep, and water buffalo) and nonruminants (e.g., horses, dogs, rabbits, and pigs) (*8*–*10*). STEC infections are transmitted to humans through contaminated food (*11*), water (*12*,*13*), and contact with infected persons (*14*) or animals (*15*,*16*).

Argentina had the highest rates of HUS globally, 10.4 and 12.2 cases/100,000 children <5 years of age in 2001 and 2002, respectively (*17*). In Argentina, HUS is the leading cause of acute renal failure among children; in 1 study, after at least 10 years of follow-up, ≈20% of Argentine children had low creatinine clearances (*5*). HUS is responsible for 20% of kidney transplants among children and adolescents in Argentina (*18*). In studies in the 1990s, evidence of STEC infection was found in 59% of Argentinean HUS case-patients, and *E. coli* O157 was the predominant serogroup (*19*,*20*). In 2000, HUS became reportable in Argentina, and sentinel sites began screening for STEC on all routine stool cultures. Given the high rate of HUS, the lack of definitive treatment, and the high morbidity, primary prevention of STEC infections is needed to lower the incidence of childhood kidney disease. However, controlled epidemiologic studies to identify risk factors associated with STEC infection have not been conducted in Argentina. To evaluate risk factors for sporadic STEC infection, we conducted a case-control study in 2 sites, Mendoza and Buenos Aires cities and their surroundings.

## Methods

### Case Ascertainment

Patients were enrolled from January 2001 through December 2002 from the public tertiary-care pediatric hospitals, Hospital “Dr. Humberto Notti” in Mendoza, which serves an urban and semirural area, and Hospital “Dr. Juan P. Garrahan” in Buenos Aires, which serves an urban area. Study personnel detected STEC cases through daily review of the hospitals’ laboratory records and detected diarrhea-associated HUS cases through biweekly discussions with nephrologists.

### Definitions

A case of STEC infection was defined as illness in a previously healthy child <16 years old who was evaluated at a participating institution and who had either culture-confirmed O157 STEC diarrhea, culture-confirmed non-O157 STEC diarrhea, or definite diarrhea-associated HUS. For convenience, we included only children permanently residing within 15 km of each institution. Local investigators stated that the characteristics of the population within each of these areas were similar to that within 50 or 100 km of the respective hospitals. We chose these 2 areas so that the population serviced would be similar to the Argentine population living in areas with a high incidence of HUS.

Definite HUS was defined as acute microangiopathic hemolytic anemia, thrombocytopenia, and renal impairment as determined by 1) hematocrit <30% with microangiopathic changes on peripheral blood smear (e.g., schistocytes, burr cells, helmet cells, or red cell fragments); 2) platelet count <150,000/mm^3^; and 3) serum creatinine concentration >2 standard deviations above the upper limit of normal for age and sex (*21*); or abnormal urinary sediment by dipstick, i.e., hematuria (>2+) or proteinuria (>2+). HUS was considered diarrhea-associated when a diarrheal illness preceded HUS by <3 weeks. We excluded children with a family history of HUS, secondary HUS (e.g., drug-associated), or HUS associated with pneumococcal infection. Probable HUS was defined as an illness that met only 2 of the laboratory criteria in a patient with culture-confirmed STEC infection.

### Case–Control Study

For each patient enrolled, 2 age- and neighborhood-matched control children without gastrointestinal illness in the 2 weeks before the matched case’s illness onset were identified. We neighborhood-matched control children to control for socioeconomic status, which was not a factor of interest, was a possible confounder, and was quite variable in the study areas. In urban areas, a trained interviewer sought controls by walking, starting at the case-patient’s home, going to the third house from the nearest corner, and continuing to every house on the block, then to the block facing the case-patient’s residence, then to other blocks in a clockwise fashion, until 2 eligible and consenting controls were interviewed. In nonurban areas, the interviewer randomly chose a cardinal direction and then sought controls beginning from the third residence from the case patient’s house in that and the opposite direction until controls were found. Informed consent was obtained from the adult primary caregiver, who was interviewed with a standardized questionnaire, administered in person. Most eligible controls were enrolled; information on potential controls who were excluded or chose not to participate was not kept.

The questionnaire (available upon request from the corresponding author) had 89 major questions and was divided into 3 major sections: characteristics of and treatment for the illness (19 questions), exposures (55 questions), and demographics (15 questions). Exposure questions were divided into sections that dealt with human contacts , care and feeding of young children, water sources and treatment, beef, other meats, fruits and vegetables, meat handling at home, animal contact, swimming, and travel. Many exposure questions had several parts (e.g., if beef consumed, was it pink; if chicken consumed, indicate if at home, restaurant, or other location). Almost all responses were measured discretely (i.e., categorically). Responses to each question were treated independently, except for those about fruits and vegetables, which were combined and dichotomized at the median; this analysis method was checked against other choices and found robust. All exposure questions for case-patients and controls were about the 7 days before the onset of illness. Interviews of case-patients were conducted a median of 12 days (range 3–41) after diarrhea onset; control interviews were conducted a median of 15 days (range 1–41) after diarrhea onset in the matched case. Controls were age-matched to case-patients by using the following groups: <12 months; 1–5 years ± 1 year; 6–9 years ± 2 years; 10–15 years ± 3 years.

### Laboratory Methods

Fecal samples were plated onto sorbitol-MacConkey agar directly, and after enrichment at 37°C for 4 h in trypticase soy broth were supplemented with cefixime (50 ng/mL) and potassium tellurite (25 mg/mL). The confluent growth zone and colonies were screened for *stx*1, *stx*2, and *rfb*O157 genes by a multiplex PCR (*22*,*23*). Isolates with *stx*1 or *stx*2 genes were identified by standard biochemical methods. *Stx*-positive colonies were serotyped (*24*) and characterized at the Argentina National Reference Laboratory (*11*,*25*).

### Statistical Analysis

Data analysis used 3 steps: an initial univariate analysis, a second univariate analysis adjusted for highly significant factors of prior or secondary interest, and a final multivariable model-building analysis. Four factors were ultimately chosen in the second univariate analysis as a fixed set from which to explore further model-building, based on epidemiologic sensibility, strong association, and stability of subsequent adjusted associations. Single- and multiple-variable conditional logistic regression models were used to evaluate associations between the outcome and exposure variables. At each step, risk factors that were statistically significant (p<0.05) and had biologic plausibility were selected for further modeling. In multivariable model-building, we pursued forward, backward, and the best subset selection strategies, as well as manual strategies. Standard methods were used to assess model fit, including residual analyses. Maximum likelihood parameter estimates from these models were used to calculate point estimates and confidence intervals for odds ratios, referred to henceforth as matched odds ratios. Exact analysis was used where small sample size would make asymptotic analysis suspect, and Mantel-Haenszel odds ratios were computed when maximum likelihood estimates did not exist.

Exploratory and sensitivity analyses were performed on subsets of the data defined by location and subcategory of disease status. Because the subsets by site (Buenos Aires and Mendoza) and by serogroup (O157, non-O157) were small and most factors examined had already been demonstrated as risky or protective in the larger dataset, we used a p value of 0.10 to assess significance by site and serogroup. For similar reasons, we did not perform multivariable analyses on these subsets. Three patients with mixed STEC infection and 2 *stx*-positive HUS patients without STEC isolated were excluded from the analysis by serogroup. Data were analyzed with Epi Info 2000 (Centers for Disease Control and Prevention, Atlanta, GA, USA) and SAS 9.0 (SAS Institute, Inc, Cary, NC, USA) software. The study was approved by the hospitals’ ethics committees as well as the institutional review boards of the Ministry of Health of Argentina and CDC.

## Results

### Case and Control Characteristics

Among 157 eligible case-patients, 150 (96%) were enrolled; the parents of 1 child refused, and interviewers could not contact the parents of 6. The hospital “Dr. Juan P. Garrahan” in Buenos Aires enrolled 54% of the cases, and the hospital “Dr. Humberto Notti” in Mendoza enrolled 46%. Among the 150 enrolled cases, 17 met both entry criteria of culture-confirmed STEC infection and definite HUS, 82 met only the criterion of culture-confirmed STEC infection, and 51 met only the criterion of definite HUS. In addition to the patients with definite HUS, 10 patients with culture-confirmed STEC infection had probable HUS.

The median age of case-patients was 1.8 years (range 4 months–14 years). The median age of the 299 controls was similar (2.0 years; range 1 month–17 years); 58% of case-patients versus 43% of controls were female (p = 0.01). One hundred and thirty-four (89%) case-patients were urban residents.

### Clinical Findings

Among the 150 case-patients, clinical findings included bloody diarrhea (84%) and vomiting (71%). Ninety-four (63%) were hospitalized for a median of 4.5 days (range 1–14 days). Among the 78 case-patients with definite HUS (dHUS n = 68) or probable HUS (pHUS n = 10), 37 (47%) had peritoneal dialysis (34 dHUS 50%, 3 pHUS 30%), 2 (3%) had hemodialysis (all dHUS), and 69 (88%) received erythrocyte transfusions (62 dHUS, 7 pHUS).

### Stool Cultures and Characterization of Isolates

Among the 99 case-patients with culture-confirmed STEC infection, 96 had a single STEC isolated: 58 (60%) were O157:H7, and 38 (40%) non-O157. Among the 99 with STEC isolated, the proportion who had O157 was 82% (14 of 17) among children with definite HUS, 90% (9 of 10) among children with probable HUS, and 53% (38 of 72) among children without HUS. Among the 38 non-O157 isolates, the serotype frequency was 29% O145:NM, 11% O26:H11, 11% O113:H21, 8% O174:H21, 5% O8:H19, 5% O145:H25, 5% ONT:NM, and 3% (1 each) O2:H11, O15:H27, O25:NM, O58:H40, O91:H7, O103:H2, O103:H25, O111:NM, O121:H19, and O171:H2.

### Risk Factors Overall

Analysis of single variable associations, when the fixed adjustment factors were controlled for, identified dietary habits and animal exposures linked to illness (Table 1). General dietary habits linked to STEC illness included eating at a social gathering, eating any meal prepared at home, and drinking from a baby bottle left at room temperature for >2 hours. Many beef-related exposures were significantly associated with STEC infection (Tables 1, 2). Eating beef outside the home and eating undercooked beef (described as uncooked, red and juicy, or pink) anyplace was associated with illness. Eleven percent of case-patients but only 5% of controls consumed *jugo de carne* (liquid squeezed from a tender, usually lightly cooked piece of beef, and spoon-fed); case-patients with this exposure ranged from 7 months to 9 years old. Living in or visiting a place with farm animals, contact with farm animals (including horses, pigs, poultry, and cattle), and contact with cattle manure were associated with illness. Risky exposures that suggest person-to-person transmission from young children included contact with a child <5 years of age, attending daycare or kindergarten, and contact with a child <5 years of age with diarrhea. Wearing diapers was also linked to illness. No significant differences between case-patients and controls were found in the distribution of most variables that relate to socioeconomic status (e.g., number of bedrooms, water supply, garbage disposal, educational level of parents). However, case households were more likely to have nonparental income.

**Table 1:** Univariate analysis of risk factors for Shiga toxin–producing *Escherichia coli* infections, unadjusted and adjusted, Buenos Aires and Mendoza, Argentina, 2001–2002

Risk factors	% Case-patients† (N = 150)	% Controls† (N = 299)	Unadjusted univariate analysis				Adjusted univariate analysis*			Sites‡
			mOR	95% C‡	p value		mOR	95% CI	p value	
General dietary habits										
Eating at a social gathering	18	8	2.79	1.4–5.3	0.002		3.77	1.8–8.1	0.0007	B, M
Eating any meal prepared at home	93	88	2.26	1.0–5.0	0.047		3.22	1.3–7.7	0.009	B
Drinking from baby bottle left at room temperature for >2 h	71	63	1.70	1.0–2.8	0.043		1.89	1.1–3.4	0.029	B
Beef-related dietary habits										
Eating beef outside home	22	15	1.70	1.0–2.9	0.06		2.18	1.2–4.1	0.014	B
Eating meatballs	3	0	0.46	0.3–0.8	0.004		15.00§	1.7–136.2	0.005	M
Eating breaded beef (*milanesa*)	3	0.3	10.00	1.2–85.6	0.036		13.45	1.4–125.0	0.022	B
Eating undercooked beef any place	29	14	2.69	1.6–4.5	0.0002		2.65	1.5–4.8	0.001	B
Eating undercooked piece of beef	19	9	2.46	1.4–4.4	0.003		2.38	1.2–4.6	0.010	B
Eating undercooked ground beef	11	5	2.41	1.1–5.1	0.021		2.70	1.1–6.5	0.026	B
Eating undercooked beef outside home	5	0.3	14.00	1.7–113.8	0.014		25.04	2.6–242.4	0.005	B
Eating undercooked beef at home	26	14	2.33	1.4–3.9	0.001		2.23	1.2–4.0	0.008	B
Teething on undercooked beef	11	2	4.83	1.9–12.4	0.001		4.00	1.4–11.4	0.010	B
Consuming *jugo de carne*¶	11	5	2.19	1.2–4.0	0.009		3.23	1.3–7.8	0.009	B, M
Eating undercooked piece of beef	18	9	2.21	1.2–4.0	0.009		2.05	1.1–4.0	0.033	B
Eating undercooked steak	13	6	2.34	1.2–4.6	0.015		2.03	0.0–4.3	0.060	B
Eating salami at home	19	11	2.19	1.2–4.0	0.009		2.22	1.1–4.5	0.027	B
Exposure to animals or their environment										
Living in or visiting a place with farm animals	13	5	3.49	1.5–7.9	0.003		4.86	1.9–12.8	0.001	B, M
Contact with farm animals	11	5	2.25	1.0–4.8	0.036		4.45	1.7–11.6	0.002	B, M
Contact with cattle manure	3	1	4.33	0.8–22.8	0.084		9.03	1.0–86.1	0.050	M
Contact with horses	10	4	2.76	1.2–6.4	0.02		5.02	1.7–14.5	0.003	M
Contact with pigs	5	2	2.13	0.7–6.2	0.20		3.80	1.0–13.4	0.041	M
Contact with poultry	6	4	1.68	0.7–4.2	0.26		2.90	1.0–8.2	0.050	M
Contact with cattle	4	2	1.92	0.6–6.5	0.29		3.51	0.8–14.7	0.085	M
Person-to-person transmission										
Contact with a child <5 y	80	67	2.08	1.3–3.4	0.003		2.05	1.2–3.5	0.009	B, M
Attending daycare or kindergarten	17	9	2.87	1.4–5.9	0.004		2.34	1.1–5.1	0.034	B, M
Contact with a child <5 y with diarrhea	15	6	3.61	1.6–8.4	0.003		2.54	1.0–6.6	0.050	M
Other variables										
Wearing diapers	72	62	2.63	1.4–5.0	0.003		2.12	1.0–4.3	0.036	–
Nonparental household income	56	40	1.89	1.3–2.8	0.002		1.98	1.2–3.2	0.005	B

*Adjusted by the fixed adjustment factors shown in Table 2. mOR, matched odds ratio; CI, confidence interval. †The denominator (number of respondents) for case-patients varied from 146 to 150, except for contact with a child <5 y with diarrhea in which the number was 119. The denominator for controls varied from 292 to 299, except for contact with a child <5 y with diarrhea in which the number was 263. ‡Denotes adjusted univariate analysis significant in Buenos Aires (B), Mendoza (M), or neither site (–). §Cochran-Mantel-Haenszel odds ratio. ¶Liquid squeezed from a tender, usually lightly cooked piece of beef, and spoon-fed.

**Table 2:** Univariate analysis of protective factors for Shiga toxin–producing *Escherichia coli* infections, unadjusted and adjusted, and adjustment factors, Buenos Aires and Mendoza, Argentina, 2001–2002

Characteristic	% Case-patients† (N = 150)	% Controls† (N = 299)	Unadjusted univariate analysis				Adjusted univariate analysis*			Sites‡
			mOR	95% CI	p value		mOR	95% CI	p value	
Protective factors										
Eating meatballs at home	17	29	0.46	0.3–0.8	0.004		0.44	0.2–0.8	0.010	M
Eating meat pie at home	11	23	0.40	0.2–0.8	0.004		0.47	0.2–0.9	0.025	–
Eating *empanadas* at home	20	34	0.43	0.3–0.7	0.001		0.49	0.3–0.9	0.016	M
Respondent always washing hands with soap and water after handling raw beef	50	64	0.53	0.3–0.8	0.004		0.57	0.3–0.9	0.019	B, M
Fixed adjustment factors										
Eating more than the median number of fruits and vegetables	33	51	0.42	0.3–0.7	0.0002		–	–	–	B, M
Male sex	43	57	0.57	0.4–0.9	0.01		–	–	–	B, M
Having a nonparent respondent	3	1	0.29	0.1–0.8	0.009		–	–	–	M
Respondent always washing hands after handling raw beef	74	90	0.27	0.1–0.5	0.0001		–	–	–	B, M

*Adjusted by the fixed adjustment factors shown. mOR, matched odds ratio; CI, confidence interval. †The denominator (number of respondents) for case-patients varied from 144 to 150. The denominator for controls varied from 298 to 299. ‡Denotes adjusted univariate analysis significant in Buenos Aires (B), Mendoza (M), or neither site (–).

Four protective factors were identified, all related to beef (Table 2). These were the child eating meatballs at home; the child eating *empanadas* (fried or baked pastries with ground beef filling) at home; the child eating meat pie at home; and the respondent always washing hands with soap and water after handling raw beef. The factors controlled for in the adjusted univariate analysis were eating more than the median number of fruits and vegetables, male sex, having a nonparent respondent, and the respondent always washing hands after handling raw beef; all were protective (Tables 1, 2).

On multivariable logistic regression analysis, significant risk factors for STEC infection that remained were eating undercooked beef outside the home (odds ratio [OR] 17.63, 95% confidence interval [CI] 1.6–197.4, p = 0.02), living in or visiting a place with farm animals (OR 6.61, 95% CI 1.5–28.8, p = 0.01), contact with a child <5 years of age with diarrhea (OR 3.29, 95% CI 1.0–10.4, p = 0.04), and having nonparental household income (OR 2.21, 95% CI 1.2–4.0, p = 0.01). Eating ground beef at home (meatballs, *empanadas*, or meat pie) remained protective (OR 0.36, 95% CI 0.1–0.9, p = 0.03). With this model, the fixed adjustment factors had significant estimated protective associations as follows: eating more than the median number of fruits and vegetables (OR 0.31, 95% CI 0.1–0.6, p = 0.0007), male sex (OR 0.34, 95% CI 0.2–0.7, p = 0.001), having a nonparent respondent (OR 0.34, 95% CI 0.2–0.7, p = 0.001), and the respondent always washing hands after handling raw beef (OR 0.23, 95% CI 0.1–0.6, p = 0.001).

### Risk Factors by Site and Etiology

We performed a univariate adjusted analysis for all variables by site (Tables 1, 2). For every variable that was significantly risky or protective in the combined analysis, the OR went in the same direction in the site-specific analysis (Buenos Aires 81 cases, Mendoza 69 cases), although the association was not always statistically significant. Many dietary habits, most of which were beef associated, were significantly associated with illness in Buenos Aires, whereas fewer reached statistical significance in Mendoza. Consuming *jugo de carne* was significantly associated with illness in both sites; however, 19.1% of case-patients from Mendoza consumed this item compared with only 4.9% from Buenos Aires.

Risk and protective factors were also analyzed separately for patients with culture-confirmed O157 or non-O157 STEC infection (Table 3). These 2 groups were similar in age, sex, and site distribution. The risk and protective factors among these 2 groups were similar to those of all study participants. Among patients with O157 STEC infection, illness was significantly associated with eating at a social gathering, with many meat-related dietary habits, exposures related to farm animals and their environment, wearing diapers, and having a nonparental household income. Protective factors included several related to eating beef at home and buying beef less than once a week. Among the smaller group of patients with non-O157 STEC infection, the only risk factors significantly linked to illness were drinking from a bottle left at room temperature, drinking formula (a factor not identified in the full group), eating a piece of beef outside the home, teething on undercooked beef at home, contact with a child <5 years of age with diarrhea, wearing diapers, and living in an overcrowded condition. Eating meat pie at home was protective for this group.

**Table 3:** Adjusted univariate analysis of risk and protective factors for Shiga toxin–producing *Escherichia coli* (STEC) O157 and non-O157 STEC, Buenos Aires and Mendoza, Argentina 2001–2002*

Characteristics	STEC O157					Non-O157 STEC			
	% Case-patients† (n = 58)	% Controls† (n = 116)	mOR	p value		% Case-patients† (n = 38)	% Controls† (n = 75)	mOR	p value
Risk factors									
Dietary habits									
Eating at a social gathering	19	8	9.79	<0.01		21	7	2.82	NS
Drinking from a baby bottle left at room temperature for >2 h	75	76	1.32	NS		66	53	3.78	<0.05
Drinking formula (milk)‡	3	3	1.91	NS		11	1	12.70	<0.05
Meat-related dietary habits§									
Eating breaded beef (*milanesa*) at restaurant	5	0	15.00¶	<0.05		3	1	2.02	NS
Eating a piece of beef outside home§	12	5	4.68	<0.05		13	1	7.56	<0.10
Eating undercooked beef at any place	29	10	3.69	<0.05		29	17	1.96	NS
Teething on undercooked beef at home	16	3	4.15	<0.10		8	1	12.78	<0.10
Consuming *jugo de carne*¶	16	4	3.24	<0.10		8	7	2.22	NS
Eating undercooked piece of beef	22	8	3.29	<0.10		16	11	1.25	NS
Eating salami at home	24	10	3.73	<0.05		11	8	1.28	NS
Eating ham‡	40	25	2.52	<0.10		16	25	0.36	NS
Eating beef soup‡	36	48	0.44	<0.10		45	40	1.31	NS
Exposure to animals or their environment									
Living in or visiting a place with farm animals	18	5	11.83	<0.01		13	5	2.76	NS
Contact with farm animals at any place	14	5	6.08	<0.05		13	7	3.39	NS
Contact with horses	12	5	4.51	<0.05		13	4	6.78	NS
Person-to-person transmission									
Contact with a child <5 y with diarrhea	20	5	6.29	NS		26	4	6.93	<0.05
Other variables									
Wearing diapers	82	70	2.83	<0.10		82	65	9.34	<0.10
Nonparental household income	58	42	2.06	<0.10		45	47	1.02	NS
Living in overcrowded condition‡	22	15	1.77	NS		31	1	3.06	<0.10
Protective factors									
Eating meat pie at home	17	23	0.77	NS		5	19	0.19	<0.10
Eating *empanadas* at home	16	37	0.17	<0.01		18	33	0.37	NS
Eating ground beef at home‡	69	82	0.21	<0.05		60	73	0.29	NS
Eating breaded beef (*milanesa*) at home	49	67	0.39	<0.05		45	56	0.77	NS
Buying beef <1 time/wk‡	76	93	0.24	<0.05		87	89	0.43	NS

*mOR, matched odds ratio; NS, not significant (p>0.10). †For STEC O157, the denominator (number of respondents) for case-patients varied from 56 to 58, except for contact with a child <5 y with diarrhea, in which the number was 51. The denominator for their controls varied from 112 to 116, except for this same factor, in which the number was 104. For non-O157 STEC, the denominator (number of respondents) varied from 37 to 38, except for contact with a child <5 y with diarrhea, in which the number was 31. The denominator for their controls varied from 73 to 75, except for this same factor, in which the number was 67. ‡All significant associations except these were also significant associations in the total dataset with 150 cases. §The term “meat” includes ground beef. ¶Liquid squeezed from a tender, usually lightly cooked piece of beef, and spoon-fed.

## Discussion

This first study of risk factors for sporadic STEC infections in Argentina demonstrates a broad range of factors associated with transmission. Undercooked beef in many forms was the most risky food. The presence of *E. coli* O157 in beef purchased in Argentina has also been demonstrated microbiologically (*25*). Beef, especially undercooked ground beef, is well recognized as a vehicle for *E. coli* O157:H7 infections (*26*). Our results also suggest that many STEC infections are acquired in the home as a result of breaches in kitchen hygiene in relation to beef; washing hands after handling raw beef, especially with soap and water, was protective. The protective effect of consuming some beef products at home is further evidence of the important role of the food preparer. Few beef-related factors were significantly risky in Mendoza, suggesting that this population may consume less undercooked beef; however, consumption of *jugo de carne*, a risky food, was much more common in Mendoza.

Whereas ground beef consumed as hamburgers is frequently implicated in North America, the spectrum of major risky beef items we identified in Argentina is wider. Other beef-based items linked to sporadic STEC infections in this study included ground beef, pieces of beef, pieces of tender “teething” beef, beef *milanesa* (breaded beef), steak, *jugo de carne*, and salami. Salami and other types of beef are uncommon causes of STEC outbreaks (*27*). Among 183 foodborne *E. coli* O157 outbreaks reported in the United States from 1982 to 2002, 41% were linked to ground beef, but only 6% to other beef items (*28*). These findings highlight the importance of conducting studies locally to determine local risk factors and corresponding control measures. The risk we demonstrated from contact with farm animals and their environment supports studies from other areas that this is an important mode of transmission (*9*,*29*–*32*). The variety of animals to which exposure conferred risk, including some which have never been directly implicated as a source of STEC infections, suggests widespread contamination of farm environments. Our data also indicate that person-to-person spread is an important mode of STEC transmission in Argentina, as evidenced by the increased risk for illness from contact with a young child with diarrhea and from attendance at a daycare or kindergarten. The neighborhood-matched study design limited the likelihood of identifying socioeconomic factors, but the finding that case households were more likely than controls to have nonparental household income suggests that they were poorer.

To our knowledge, this is the largest study of risk factors for sporadic non-O157 STEC infection. Most exposures that were risky for the 150 case-patients also had high ORs for the non-O157 STEC case-patients, suggesting that similar exposures are risky; however, few of the risks were statistically significant in the subgroup. This finding may partially reflect the small size (38 cases) and diversity (14 serogroups) of the non-O157 STEC subgroup. Others have also reported outbreaks and sporadic cases of non-O157 STEC infections caused by cattle-related items (*1*,*9*). However, a study from Belgium of both O157 and non-O157 STEC infection found that consumption of fish but not beef was risky (*33*). Our finding that drinking infant formula was risky only in the non-O157 subgroup merits further study. Powdered infant formula is a known source of invasive infections in infants (*34*). With larger studies, strains that are less likely to be pathogens can be excluded and serotype-specific risk factors can be examined.

To our knowledge, others have not reported male sex as a protective factor (or female sex as a risk factor) for STEC infection. However, others have reported female sex as a risk factor for *E. coli* O157–associated hemolytic anemia (*35*). The reason for this sex difference is not known. We do not know why having a nonparent responder to the questionnaire was associated with a lower risk for STEC infection. However, it suggests a setting in which help with childcare is available from family members or paid care providers.

Our finding that eating a wider variety of fruits and vegetables was protective against STEC infection merits further investigation. A varied diet may increase resistance to disease by providing bowel flora that help to protect against colonization with pathogens by providing compounds that block bacterial adhesions, as has been postulated for urinary tract infections (*36*), or by some other mechanism (*37*).

Our study had several limitations. First, we included as STEC cases children with diarrhea-associated HUS who did not have laboratory confirmation of STEC infection. However, other data indicate that almost all diarrhea-associated HUS cases in children are due to STEC infection (*7*). To our knowledge, *Shigella dysenteriae* type 1, the only other known cause of diarrhea-associated HUS (*38*), has not been isolated from ill persons in Argentina in recent decades. Inclusion of these HUS cases provided power needed for the analysis; a subanalysis examining these cases alone indicates that they did not introduce any extraneous associations into the analysis of the full dataset (data not shown). Second, we analyzed multiple exposures, which can lead to finding associations by chance alone. However, the factors we identified are plausible biologically and supported by other evidence. Third, features inherent to the study design may have led to finding risk and protective factors that were not representative of the Argentine population. Although the study population included those with urban, suburban, and rural residences, our inclusion of patients only within 15 km of study hospitals likely resulted in exclusion of some very rural segments of the population. Fourth, our neighborhood matching of controls may have led to overmatching on some fixed environmental features, but decreased the chance of identifying risk factors that were surrogates for differences in socioeconomic factors. We matched for neighborhood to control for socioeconomic status both to decrease the number of significant factors that were difficult to change and because we expected most of the causal pathways to relate to food and animal exposures. Fifth, we studied 2 geographically separated populations. Our subanalysis indicated that some risky factors were more prominent in 1 location. Studies of other Argentine populations may identify risky practices important in those populations.

We considered using the multivariable model as the complete basis for describing our results. However, we were able to create many multivariable models of similar strength that varied in the factors that remained significant. In all multivariable models, some important factors dropped out, but those factors varied. Because no one multivariable model adequately described the findings, we chose to present both the multivariable model that had the strongest individual predictors and the adjusted univariate analysis. The latter retains some important factors amenable to intervention, such as eating undercooked beef at home.

Measures are needed to decrease the likelihood of persons in Argentina consuming food contaminated with STEC. Effective safety practices at all stages of the food chain must be ensured. In particular, the contamination of beef by STEC O157 should be reduced. Major efforts to educate the Argentine public and the food industry could help to reduce these serious illnesses. Social research is needed to better understand practices involving giving meat for teething and *jugo de carne* to young children. Ensuring that beef is well cooked is a key message. Education is needed to explain the risks related to exposure to farm animals and the ability of people to protect themselves by washing hands.

Evidence indicates that measures instituted by industry, in response to government regulations, recalls, and outbreak investigations, are critical in decreasing STEC infections. After a large outbreak due to ground beef in 1993 in which 4 children died (*39*), the US Department of Agriculture declared *E. coli* O157:H7 an adulterant in ground beef; retail beef from lots known to contain the organism must now be recalled. In 2002, a recall of >18 million pounds of ground beef with *E. coli* O157 contamination (*40*), and a new USDA directive (*41*), galvanized the US beef industry to institute more aggressive pathogen control measures, including testing of all lots of beef trimmings or ground beef for *E. coli* O157 in plants (R. Huffman, American Meat Institute Foundation, pers. comm.). Implementation of prevention measures by industry, government, and consumers could result in a decrease in the incidence of STEC infections in children, and thereby decrease the incidence of childhood kidney disease from HUS in Argentina with its associated human and economic costs.
